# A novel multi-reaction microdroplet platform for rapid radiochemistry optimization†

**DOI:** 10.1039/c9ra03639c

**Published:** 2019-07-01

**Authors:** Alejandra Rios, Jia Wang, Philip H. Chao, R. Michael van Dam

**Affiliations:** Crump Institute of Molecular Imaging, University of California Los Angeles (UCLA) Los Angeles CA USA mvandam@mednet.ucla.edu; Physics and Biology in Medicine Interdepartmental Graduate Program, UCLA USA; Department of Bioengineering, UCLA USA; Department of Molecular & Medical Pharmacology, David Geffen School of Medicine, UCLA USA

## Abstract

During the development of novel tracers for positron emission tomography (PET), the optimization of the synthesis is hindered by practical limitations on the number of experiments that can be performed per day. Here we present a microliter droplet chip that contains multiple sites (4 or 16) to perform reactions simultaneously under the same or different conditions to accelerate radiosynthesis optimization.

## Abstract 2

Multi-reaction microdroplet chip enables rapid radiotracer optimization for positron emission tomography.

## Graphical abstract

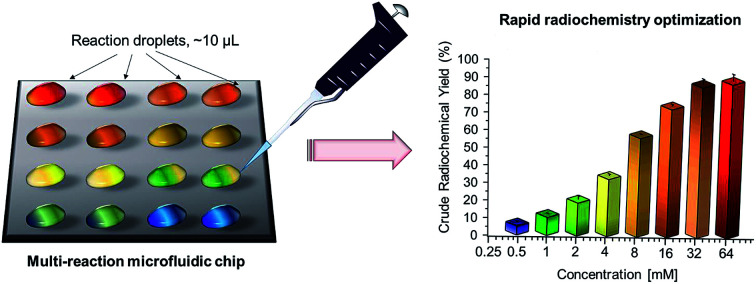

Most positron emission tomography (PET) tracers available for preclinical and clinical research are produced using automated radiosynthesizers, which facilitate consistent production and protect operators from radiation.^1,2^ Current systems are designed for production of relatively large batches that are divided up among many end users to share the production cost. While suitable for commonly used tracers (*e.g.* [^18^F]FDG), the systems can be wasteful for production of smaller batches of less commonly used tracers, or production of novel tracers for early-stage development. Reaction volumes are typically in the ∼1 mL range with needed amounts of expensive precursors in the range of 1–10s of mg, and the systems must be operated in specialized hot cells to provide radiation protection.

The issue is especially apparent during optimization of synthesis protocols to achieve sufficient and consistent yield. Using conventional radiosynthesizers, optimization is cumbersome because the apparatus becomes contaminated after use and one must wait for radioactive decay (*e.g.* overnight) before beginning the next experiment. The limited experimental throughput extends studies over weeks or months and has significant associated cost, including labor, facilities, multiple batches of radionuclide, and high amount of precursor needed per reaction. These challenges hinder the development of new tracers and limit the progress of research using those tracers.

Recently, Zhang *et al.* reported a high-throughput technique for optimization of ^18^F-radiosyntheses^3^ that avoids the use of radioactivity, thus allowing multiple syntheses to be carried out back to back on the radiosynthesizer without worry of radioactive contamination of the apparatus. Syntheses are performed starting with levels of [^19^F]fluoride (in the form of KF) that are comparable to what would be expected in an actual radiosynthesis (using [^18^F]fluoride), and reaction yield is determined by detecting species of interest in the crude reaction mixture with very high sensitivity using liquid chromatography/tandem mass spectrometry (LC-MS/MS). Reasonable correlation of yields between the non-radioactive approach and conventional radiosynthesis were reported for two PET tracers, [^18^F]fallypride and [^18^F]MDL100907.^3^ While enabling optimization to be carried out in a shorter time and reducing radionuclide costs, this technique relies on a very expensive instrument that is not commonly found in radiochemistry laboratories. Furthermore, the optimization remains labor-intensive as reactions are carried out serially.

In recent years, microfluidic radiochemistry has drawn increasing attention due to several advantages over radiochemistry performed in conventional radiosynthesizers.^4,5^ Several microfluidic platforms have demonstrated reactions in very small volumes and with short synthesis times,^5–10^ yet with comparable radiochemical yield to conventional approaches. As a result of the small volume, consumption of expensive reagents (*e.g.* precursors, peptides, proteins…) can be orders of magnitude less,^10^ purification can be simplified and accelerated, and high molar activity of the tracer can be achieved, even when using only a small amount of radioactivity.^11^ All these factors contribute to significant reductions in the cost of radiosynthesis, and have particular impact when only small batches are needed.

Leveraging the benefits of microfluidic radiochemistry, Pascali *et al.* reported an optimization protocol for ^18^F-radiosyntheses using a flow-chemistry based radiosynthesizer (Nanotek, Advion, Ithaca, NY, USA). Operating in a special back-to-back experiment mode, thorough optimization of radiofluorination conditions (reaction temperature, residence time and reagent ratio) could be completed in only 5–10 experimental days, which is significantly shorter than the time typically required for optimization on conventional systems.

Inspired by these advances, we developed a high throughput radiochemistry optimization platform, adapted from droplet-based microdroplet reactors developed by our group,^9^ where multiple reactions can be performed in parallel instead of sequentially. This approach uses a microdroplet chip that contains an array of reaction sites (either 2 × 2 or 4 × 4) for performing simultaneous droplet-based radiosyntheses. This technique has considerable advantages: (i) reactions are performed in parallel, with up to 16 reactions (different conditions and/or replicates) completed in the time taken to perform 1 reaction; (ii) each reaction consumes minimal reagents (typically 10s of μg), reducing the cost of optimization and enabling optimization even in early stage development when there is limited supply of the precursor; (iii) reactions are analyzed using standard analytical radiochemistry techniques and does not require significant new instrumentation. Furthermore, the platform significantly relieves the radiochemist from tedious and repetitive work. As a proof of concept, we optimize the synthesis of [^18^F]fallypride, a PET tracer used to study diseases associated with the dopaminergic system such as Parkinson's, Huntington's, and Alzheimer's diseases,^12,13^ to maximize yield, and show that an extensive optimization could be performed within a few days.

The microdroplet reaction chips had either 2 × 2 arrays of reaction sites (4 mm diameter, 9 mm pitch) or 4 × 4 arrays of sites (3 mm diameter, 5 mm pitch). The chips were fabricated by coating silicon wafers with 130 nm of (hydrophobic) Teflon and etching away the Teflon to expose the (hydrophilic) silicon surface to form the reaction sites, which act as “hydrophilic traps”. (Full details are described in ESI Section 2.†) The chips were installed on top of a heater such that the temperature was the same at all reaction sites (confirmed with thermal imaging, data not shown). The chips and overall setup are shown in Fig. 1. Syntheses were carried out in parallel, with the whole chip (*i.e.* whole array of sites) heated or cooled simultaneously after adding the relevant reagent to all reaction sites (Fig. 2). After completion of reactions, crude reaction products were collected independently from each reaction site for analysis. The detailed droplet synthesis protocol is described in the ESI, Section 3.†

**Fig. 1:**
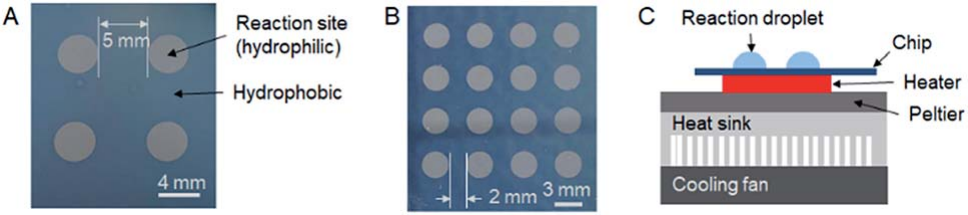
(A) Photograph of the 2 × 2 microdroplet chip. Diameter of each reaction site is 4 mm and the pitch is 9 mm. (B) Photograph of the 4 × 4 microdroplet chip. Diameter of each reaction site is 3 mm and the pitch is 5 mm. (C) Schematic of the side view of the experimental setup for performing parallel radiosyntheses on the multi-reaction chip. Up to 4 reactions can be performed in parallel on 2 × 2 array chips and up to 16 reactions can be performed in parallel on 4 × 4 array chips.

**Fig. 2:**
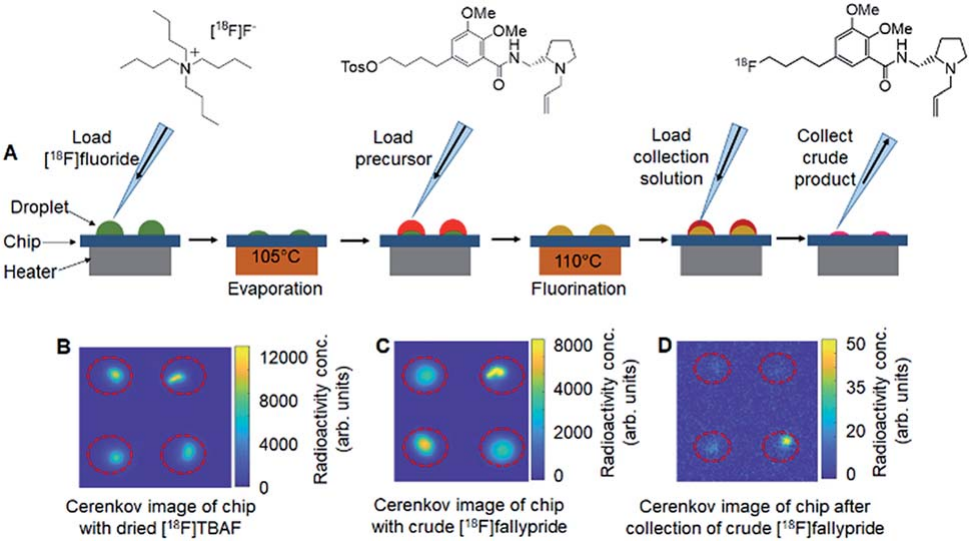
Parallel synthesis of [^18^F]fallypride using the multi-reaction platform. (A) Schematic of the multi-step radiochemical synthesis of [^18^F]fallypride in micro-droplets. First, at each site, an 8 μL droplet of [^18^F]fluoride (∼3.7 MBq) mixed with TBAHCO_3_ (240 nmol) was added and then dried at 105 °C for 1 min. Then, a 6 μL droplet of tosyl-fallypride precursor (39 mM) in 1 : 1 v/v thexyl alcohol/MeCN was added and reacted for 7 min at 110 °C. Finally, 20 μL collection solution (90 : 10 v/v MeOH : water) was loaded on the reaction site to dissolve resulting compounds and the mixed droplet was collected from the chip. Each site was independently collected for analysis *via* 3 repeats of the collection process. (B) Cerenkov image showing the distribution of radioactivity on a 2 × 2 chip (same conditions at all sites) after the evaporation of 8 μL droplets of [^18^F]fluoride mixed with TBAHCO_3_. (C) Cerenkov image showing the distribution of radioactivity of crude [^18^F]fallypride after the fluorination step. (D) Cerenkov image showing the distribution of the residual radioactivity on the chip after collection of the crude [^18^F]fallypride. Brightness is decay-corrected to a common timepoint for all images.

Evaluation of synthesis performance was achieved by analyzing the collection efficiency and fluorination efficiency to calculate the crude radiochemical yield (crude RCY). Collection efficiency was determined by dividing the activity of the collected crude sample (decay-corrected) from the starting activity in the reaction site. Fluorination efficiency was analyzed *via* radio-TLC or radio-HPLC. Complete descriptions of analytical approaches are found in the ESI Section 4.†

To assess the suitability of the multi-reaction chips for optimization, we first assessed the independence of each reaction site by performing droplet radiochemical syntheses of [^18^F]fallypride at some sites on the chip while other sites were left “blank” (no [^18^F]fluoride added, but otherwise synthesis steps still carried out). Cerenkov luminescence imaging (CLI) of the chip surface^9,14^ was used to quantify any cross-contamination of radioactive species to the blank sites at different stages of the synthesis process. In one experiment on a 2 × 2 chip, 1 of 4 sites was used to perform the first step of [^18^F]fallypride synthesis (*i.e.*, drying of solution containing [^18^F]fluoride and TBAHCO_3_ to form the [^18^F]TBAF complex), and Cerenkov images taken afterwards (ESI, Fig. S1A†) revealed negligible signal in the blank sites, *i.e.* activity level was <0.3–0.6% of the activity at the non-blank site suggesting negligible cross-contamination of radioactivity. In another experiment on a 2 × 2 chip, 3 of 4 sites were used to perform the complete synthesis of [^18^F]fallypride while a mock synthesis (no [^18^F]fluoride) was performed at the remaining site. In this case, Cerenkov images taken afterwards (ESI, Fig. S1B†) also showed negligible radioactive contamination of the blank site (<0.4%). Similarly, no significant cross-contamination was observed on 4 × 4 chips, despite the closer spacing of reaction sites. Quantitation of Cerenkov images (ESI, Fig. S2A and B†) showed the amount of contamination in blank spots to be negligible (<1.4%). Overall, these results suggest that the parallel reactions can be considered independent.

Next, we assessed the reproducibility at different reaction sites by performing replicates of syntheses using multiple reaction sites on a single chip. In a set of experiments on 2 × 2 chips, we performed drying of the [^18^F]TBAF complex and subsequent fluorination of tosyl-fallypride on all sites (ESI, Table S1†) and found the crude RCY to be 88 ± 1% (*n* = 4), indicating excellent reproducibility from site to site. Similar reproducibility was found for an experiment on a 4 × 4 chip, in which syntheses on half of the sites were carried out with a TBAHCO_3_ amount of 240 nmol, and the other half were carried out with 7 nmol (ESI, Table S2†). The crude RCYs were measured to be 85 ± 2% (*n* = 8) and 38 ± 4% (*n* = 8) for the two conditions, respectively, the low standard deviation across each condition indicates excellent site-to-site reproducibility. In later experiments (described below), we discovered that the yield is highly sensitive to the amount of base at the low-base condition, and thus the higher variability in crude RCY of those reactions is expected.

To demonstrate the utility of the platform, we then leveraged the parallel reactions to perform an extensive, fine-grained optimization of several [^18^F]fallypride synthesis parameters, each data point with multiple replicates. The initial syntheses were performed using the reaction conditions adapted from Wang *et al.*^9^ to gather baseline performance. The adapted protocol used 30 nmol of TBAHCO_3_ and a 4 μL droplet of tosyl-fallypride precursor (77 mM). In repeated experiments under identical conditions, we observed high variability of crude RCY from 38–84%, suggesting the reactions were either highly sensitive to certain conditions (*e.g.* reagent amount) or to a variable we had not accounted for.

We first explored the impact of the amount of TBAHCO_3_ in the reaction (Fig. 3A, and ESI, Table S3†). Standard deviations of data points were small, and the yield showed a clear dependence on the amount of base. From nearly zero yield at low base amount, the yield sharply rises to ∼86% at ∼80 nmol of base, where it remains relatively stable, and then falls off again with higher base amounts. The highest yield (92 ± 1%, *n* = 2) was obtained at 240 nmol. The very high sensitivity to base at 30 nmol may suggest why high variability was observed under the original synthesis conditions: a small variation in the amount of base (*e.g.* due to pipetting error when adding the [^18^F]fluoride/TBAHCO_3_ solution) could result in large variation in yield. The relatively low slope in the 80–240 nmol range suggests the yield would be fairly immune to pipetting errors.

**Fig. 3:**
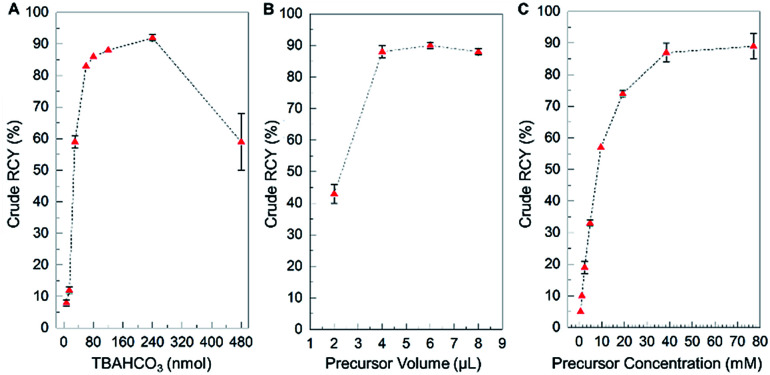
Influence of reaction parameters on the performance of the microdroplet synthesis of [^18^F]fallypride, explored using the high-throughput platform. (A) Effect of concentration of base solution. Reaction volume: 4 μL. Precursor solution concentration: 77 mM. The optimal value was taken as 240 nmol of TBAHCO_3_, with crude RCY of 92 ± 1% (*n* = 2). (B) Effect of volume of precursor solution. Base amount: 240 nmol. Precursor solution concentration: 77 mM. The optimal value was taken as 6 μL, with crude RCY of 90 ± 1% (*n* = 4). (C) Effect of concentration of the precursor solution. Base amount: 240 nmol. Precursor solution volume: 6 μL. The optimal value was taken as 39 mM, with crude RCY of 87 ± 3% (*n* = 2).

We next examined the effect of fluorination reaction volume on yield, using 240 nmol of TBAHCO_3_ in the initial [^18^F]fluoride/TBAHCO_3_ droplet and 77 mM concentration of precursor solution (Fig. 3B, and ESI, Table S4†). The crude RCY yield showed a strong dependence on reaction volume, rising from a moderate value (43 ± 3%, *n* = 4) for a 2 μL reaction to nearly 100% for volumes of 4, 6, and 8 μL. Based on visual observations, we suspect that the smaller volumes are not sufficient to fully wet the reaction site and thus some of the dried [^18^F]TBAF residue remaining after the drying step does not get dissolved into the reaction droplet. We chose a reaction volume of 6 μL for subsequent experiments as in that region the flat slope of the graph indicates an insensitivity to errors in precursor droplet volume.

Finally, we explored the influence of precursor concentration, when using 240 nmol of TBAHCO_3_ and a 6 μL fluorination reaction volume (Fig. 3C, and ESI, Table S5†). Crude RCY was near zero for low precursor concentrations, increasing rapidly with precursor concentration, and reaching a plateau with near 100% yield above ∼40 mM. At the optimal conditions (240 nmol TBAHCO_3_, 6 μL reaction volume, and 39 mM precursor concentration), the fluorination efficiency was 96.0 ± 0.5% (*n* = 2) and crude RCY was 87 ± 3% (*n* = 2).

The optimized reaction conditions found using our multi-reaction microdroplet chip provided higher and more consistent crude RCY compared to previous reports using microscale platforms.^15–18^ For example, 84 ± 7% (*n* = 6) was reported for droplet-based reactions on an EWOD chip^15^ and 64 ± 6% (*n* = 4) was reported for droplet-based reactions on a chip using a passive droplet transport mechanism.^9^ Furthermore, we were able to perform 16 syntheses within only 90 min (starting from the loading of [^18^F]fluoride/TBAHCO_3_ mixture, up to the end of collection process). On other microscale platforms, the time for a single synthesis run was, *e.g.*, 31 min ^15^ or 25 min,^9^ which would require ∼500 min [8.3 h] or ∼400 min [6.7 h] to perform 16 experiments. The time savings using the multi-reaction chip are a direct result of performing many of the steps (*e.g.* drying step and fluorination step) at all reaction sites in parallel. Interestingly, we observed the formation of a side-product on the TLC chromatogram when the molar ratio of base to precursor exceeded ∼1.0. (This observation seems to be consistent with Moon *et al.* who reported that the use of high base concentration (either K_222_/K_2_CO_3_ or TBAHCO_3_) led to low radiochemical yield and unidentified radio-impurities.^19^) We found no detectable side product as long as the molar ratio of base to precursor remained < ∼1.0 (ESI, Fig. S3†). This finding may be useful in other synthesizer setups to help choose an appropriate precursor amount depending on the amount of base needed to elute the [^18^F]fluoride from the QMA cartridge, and the ability to perform fine-grained optimization could provide critical guidance in the synthesis of other base-sensitive tracers.

Using the reaction array chips, the synthesis conditions could be rapidly optimized, and the optimization could be performed with fine granularity while including replicates of each data point. Using the 2 × 2 reaction chips, it was possible to run 16 experiments per day at low activity levels, allowing the full optimization study reported here (20 conditions, *n* = 2 each) to be completed in 3 days. By using 4 × 4 reaction chips that we started developing near the end of this study, it would be practical to complete this study in even shorter time. Further increase in throughput could be accomplished by operating multiple heaters (and multiple chips) in parallel.

Though in this study we examined the effect of reaction volume and reagent concentrations, one could also study variables such as reaction temperature or time, by using multiple heaters, or by running multiple chips sequentially on the same heater.

An important aspect of high-throughput reaction optimization is the ability to rapidly analyze all of the collected reaction mixtures. To accomplish this, we developed an optimized TLC separation method with short separation length (35 mm), and spotted multiple samples (1.0 μL each, 1.0 mm pitch) that could be separated and read out in parallel using CLI^14^ (see ESI, Section 4†).

Due to limitations of conventional radiochemistry systems that allow only one or a small number of reactions per day, one typically explores only a small range of the potential parameter space and results are often reported with no repeats (*n* = 1). Compared to such approaches, the multi-reaction droplet radiosynthesis platform makes it practical to perform more comprehensive and robust studies of radiosynthesis conditions, potentially enabling new insights on parameters that influence product yield and side-product formation, or on what choice of parameter values leads to the most robust synthesis (*i.e.* insensitivity to small variations in variables). Furthermore, since the amount of precursor consumed per reaction is extremely small (*e.g.* ∼84 μg per data point here compared to 4 mg per data point in conventional reactions), and many reactions can be carried out using the same batch of radioisotope, the cost of the optimization process can be significantly lower than for conventional setups. The low precursor consumption may be especially useful in the early development of novel tracers when only a small amount of precursor may be available. Furthermore, consistent with the concept of green chemistry,^20^ consumption of hazardous solvents is also reduced by more than 2 orders of magnitude for each microdroplet reaction, as is the generation of waste products.

Compared to optimal macroscale approaches (*e.g.* Moon *et al.*; RCY = 68 ± 2% (*n* = 42)), the optimized droplet method, combined with purification, resulted in a higher RCY of 78%.^21^ A detailed comparison of conditions is included in the ESI, Section 8.† While some differences exist between microscale and macroscale reaction volume, geometry, and heat transfer, we anticipate that the general optimization trends learned from microscale reactions could be applied to macroscale apparatus.

Due to success in synthesizing other tracers on this and similar microfluidic platforms,^9,15,22^ we expect this platform to be applicable to the development and optimization of a wide range of PET tracers and other radiopharmaceuticals. In other work, we have shown the ability to increase the scale of droplet-based reactions by pre-concentrating the radioisotope,^23^ providing a route to immediately transition from low-activity optimization runs to high-activity production runs using the exact same droplet reaction geometry and synthesis process. Thus, microdroplet reactions are not only a useful tool during the optimization phase, but also can produce sufficient quantity of tracers for preclinical or even clinical studies.

In summary, we have developed a general platform and strategy for the rapid optimization of PET tracer syntheses and demonstrates efficient translation of macroscale synthesis procedures to microscale syntheses by using a novel multi-reaction microdroplet chip that allows analysis of up to 16 parallel reactions. Contamination tests confirmed the independence of reaction sites and reproducibility of reactions was demonstrated by performing replicate syntheses.

We thank the UCLA Biomedical Cyclotron Facility for generously providing [^18^F]fluoride for these studies. This work was supported in part by the National Cancer Institute (R21 CA212718), the National Institute of Mental Health (R44 MH 097271), and the National Institute of Biomedical Imaging and Bioengineering (T32 EB002101). The authors thank the UCLA Integrated Systems Nanofabrication Cleanroom at the California NanoSystems Institute for the use of instruments for chip fabrication.

## Author contribution

A. R. and J. W. performed experiments and analyzed data. P. H. C. assisted with experiments involving base concentration. A. R., J. W., and R. M. V. contributed to the experimental design. A. R., J. W. and R. M. V. wrote the manuscript. R. M. V. supervised the project. All authors have edited the manuscript and approved the final version.

## Conflicts of interest

The Regents of the University of California have licensed technology to Sofie, Inc. that was invented by Dr van Dam, and have taken equity in Sofie, Inc. as part of the licensing transaction. Dr van Dam is a founder and consultant of Sofie, Inc. The remaining authors declare no conflicts of interest.

## Supplementary Material

RA-009-C9RA03639C-s001
