# Supplementary material for: A novel multi-reaction microdroplet platform for rapid radiochemistry optimization
Source: RSC Adv. 2019 Jul 1;9(35):20370–4. doi: 10.1039/c9ra03639c (PMC9065505; doi:10.1039/c9ra03639c)
Supplement: RA-009-C9RA03639C-s001 [file RA-009-C9RA03639C-s001.pdf]

Electronic Supplementary Material (ESI) for RSC Advances.
This journal is © The Royal Society of Chemistry 2019

Supplemental Information

A novel multi-reaction microdroplet platform for rapid radiochemistry optimization

Alejandra Rios[1,2,#], Jia Wang[1,3,#], Philip H. Chao[1,3], R. Michael van Dam[1,2,3,4,*]

[1] Crump Institute for Molecular Imaging, University of California at Los Angeles (UCLA), Los Angeles, CA, USA

[2] Physics in Biology and Medicine Interdepartmental Graduate Program, UCLA

[3] Department of Bioengineering, UCLA

[4] Department of Molecular & Medical Pharmacology, David Geffen School of Medicine, UCLA

* Corresponding author: mvandam@mednet.ucla.edu

[#] Contributed equally to this work

Table of Contents

1. **MATERIALS** 2
2. **MULTI-REACTION MICRODROPLET CHIPS** 2
3. **SYNTHESIS AND OPTIMIZATION OF [^{18}F]FALLYPRIDE ON THE CHIP** 2
4. **ANALYTICAL METHODS** 3
5. **CROSS-CONTAMINATION AND REPEATABILITY STUDIES** 4
6. **OPTIMIZATION DATA** 7
7. **HPLC CHROMATOGRAM OF CRUDE [^{18}F]FALLYPRIDE** 9
8. **COMPARISON TO MACROSCALE SYNTHESIS** 9
9. **REFERENCES** 11

1 Materials

Teflon AF 2400 (1% solids) solution was purchased from Chemours (Wilmington, DE, USA). Positive photoresist (MEGAPOSIT SPR 220-7.0) and developer (MEGAPOSIT MF-26A) were purchased from MicroChem (Westborough, MA, USA). Additional solvents and chemicals used for microfluidic chip fabrication, including methanol (MeOH, Cleanroom LP grade), acetone (Cleanroom LP grade) and isopropanol (IPA, Cleanroom LP grade) were purchased from KMG Chemicals (Fort Worth, TX, USA).

Anhydrous methanol (MeOH, 99.8%), anhydrous acetonitrile (MeCN, 99.8%), 2,3-dimethyl-2-butanol (thexyl alcohol, 98%), ammonium formate (NH_4HCO_2, 97%) and trimethylamine (TEA, 99%) were purchased from Sigma-Aldrich (St. Louis, MO, USA). Tetrabutylammounium bicarbonate ($TBAHCO_3$, 75mM), tosyl fallypride (fallypride precursor, >90%) and fallypride (reference standard for [^{18}F]fallypride, >95%) were purchased from ABX Advanced Biochemical Compounds (Radeberg, Germany). DI water was obtained from a Milli-Q water purification system (EMD Millipore Corporation, Berlin, Germany). No-carrier-added [^{18}F]fluoride in [^{18}O]H_2O was obtained from the UCLA Ahmanson Biomedical Cyclotron Facility.

2 Multi-reaction microdroplet chips

The multi-reaction microfluidic chips were fabricated utilizing 4" silicon wafers (P type, boron doped, thickness 525 ± 25 µm; Silicon Valley Microelectronics Inc., Santa Clara, CA, USA) as substrates and standard lithographic processes reported previously[1]. The 4-spot chip (25.0 x 27.5 mm^2) comprises a hydrophobic Teflon surface with four circular hydrophilic silicon reaction sites (4 mm diameter) positioned in 2 x 2 array with 5 mm space between adjacent reaction sites (9 mm center to center). The 16-spot chips (25.0 x 27.5 mm^2) have sixteen circular hydrophilic reaction sites (3 mm diameter) positioned in a 4 x 4 array with 2 mm space in between (5 mm center to center). The Teflon coating thickness was 127 ± 6 nm (n=4), determined using a stylus profiler (Dektak 150 Surface Profiler, Plainview, NY, USA).

Though one might think of each spot as a cylindrical "well" (with volume ~1.6 µL for each 4 mm diameter spot on the 2x2 chips, and ~0.90 µL for each 3 mm diameter spot on the 4x4 chips), each reaction site can actually hold significantly more volume by acting as a "hydrophilic trap". Depending on the properties of the liquid, droplets up to ~40 µL could be loaded into the 4 mm diameter sites without overflowing onto the surrounding hydrophobic region.

The chip was affixed to a temperature control platform, which was previously described[1].

3 Synthesis and optimization of [^{18}F]fallypride on the chip

[^{18}F]Fallypride was synthesized using a modified version of a previously described droplet synthesis protocol [1]. Briefly, a [^{18}F]fluoride stock solution (30 mM $TBAHCO_3$; 4 mCi; 148 MBq) was prepared by mixing with $TBAHCO_3$ with [^{18}F]fluoride/[^{18}O]H_2O and diluting with DI water up to 150 µL. An 8 µL droplet of this stock solution was loaded to each of the desired spot(s) on a 2 x 2 or a 4 x 4 multi-reaction chip and dried for 1 min at 105 °C. Next, a 6 µL solution of fallypride precursor (39 mM in 1:1 v/v mixture of thexyl alcohol and MeCN) was added, mixed with the dried residue at the desired spot(s), and allowed to react for 7 min at 110 °C. Crude [^{18}F]fallypride product was collected from the desired spot(s) on the chip, with 60 µL of 90% MeOH and 10% DI water.

Variation of individual parameters ($TBAHCO_3$ concentration, volume of precursor solution, and precursor concentration) was carried out with at least n=2 replicates each to determine their influence on fluorination efficiency and crude radiochemical yield (RCY). Volume of precursor was varied from 2 to 8 µL, $TBAHCO_3$ concentration was varied from 0.95 to 60 mM, and precursor concentration ranged from 0.6 to 77 mM.

4 Analytical methods

The activity distribution on the chips at three different stages of [^{18}F]fallypride synthesis (after drying of fluoride, after fluorination, and after collection) were analyzed using Cerenkov imaging as previously described[1,2]. Briefly, chips were placed in a home-built light-tight box[2], covered with a transparent substrate, and Cerenkov light was detected by a scientific cooled camera (QSI 540, Quantum Scientific Imaging, Poplarville, MS, USA) equipped with a 50 mm lens (Nikkor, Nikon, Tokyo, Japan). The temperature of the camera was maintained at -10°C for dark current reduction and the field of view of the system was 50 x 50 mm^2. Exposure time was 5 min and raw images were corrected as previously described[2], followed by background subtraction and decay correction to the starting time of the first image. The images were then analyzed by drawing regions of interest (ROIs) using custom-written MATLAB software. (One of the ROIs, drawn in an area that did not contain radioactive sample, was used for background subtraction.)

Moreover, performance of synthesis was evaluated by analyzing the collection efficiency and fluorination efficiency to obtain the crude radiochemical yield (crude RCY). Radioactivity measurements were made using a calibrated dose calibrator (CRC-25R, Capintec, Florham Park, NJ, USA). Activity on the chip was first measured after adding an 8 µL droplet mixture of $TBAHCO_3$ with [^{18}F]fluoride/[^{18}O]H_2O to the first reaction site and subsequent measurements were performed after adding activity to each reaction site on the chip. Subtraction and decay correction to the first measurement on the chip was performed to calculate the starting activity on each reaction site. Collection efficiency was determined by dividing the activity of the collected crude sample (decay corrected) by the starting activity in the reaction site. Fluorination efficiency was analyzed using radio-TLC. The crude sample was spotted (1 µL) onto a silica gel 60 F_{254} plate (Merck KGaA, Darmstadt, Germany), and developed with 60% MeCN in 25 mM NH_4HCO_2 with 1% TEA (v/v). To accelerate analysis, radio-TLC plates were spotted with multiple samples (up to 8 at 0.5 mm pitch) before developing. After separation, a glass microscope slide (76.2 mm x 50.8 mm, 1 mm thick) was placed over the multi-sample plates and were read out using Cerenkov imaging (5 min exposure)[3] using the same method as for imaging the microfluidic chips. To determine the fluoride conversion, ROIs were drawn on the final corrected image to enclose the radioactive regions/spots. Each ROI was integrated, and then the fraction of the integrated signal in that ROI (divided by the sum of integrated signal in all ROIs corresponding to the particular sample) was computed. Finally, crude RCY was determined by multiplying the collection efficiency by the fluorination efficiency.

Analytical radio-HPLC was performed using a Smartline HPLC system (Knauer, Berlin, Germany) equipped with a degasser (Model 5050), pump (Model 1000), a UV (254nm) detector (Eckert & Ziegler, Berlin, Germany) and a gamma-radiation detector and counter (B-FC- 4100 and BFC-1000; Bioscan, Inc., Poway, CA, USA). Separation was performed using a C18 column (Kinetex, 250 x 4.6 mm, 5 µm, Phenomenex, Torrance, CA, USA). The mobile phase was 60% MeCN in 25 mM NH_4HCO_2 with 1% TEA (v/v) and flow rate was 1.5 mL/min. The retention time of fallypride was 4.5 min. The crude [^{18}F]fallypride mixture collected from the chip was mixed with fallypride (reference standard) and manually injected into the HPLC system for confirmation of radiochemical identity.

5 Cross-contamination and repeatability studies

To investigate the cross contamination between adjacent reaction sites, Cerenkov luminescence imaging (CLI) was employed to image the activity distribution on the chip after performing drying of [^{18}F]fluoride/$TBAHCO_3$ or fluorination in different patterns of reaction sites. In an initial test of 2x2 chips, an 8 µL droplet of [^{18}F]fluoride/$TBAHCO_3$ solution (~1.8MBq; 3.8 mM) was loaded on 1 of 4 reaction sites, 8 µL droplets of $TBAHCO_3$ solution (3.8 mM) were loaded on the remaining reaction sites, and all spots were dried simultaneously at 105 °C for 30 s, followed by CLI imaging (**Figure S1A**). Suspecting that steps with more volatile organic solvent could increase cross-contamination, we also performed investigations during the fluorination reaction. On a 2x2 chip, 3 of 4 sites were loaded with 8 µL of [^{18}F]fluoride/$TBAHCO_3$ (~1.8 MBq; 3.8 mM), while one was loaded with just $TBAHCO_3$ solution (no [^{18}F]fluoride). After the drying step, 6 µL of 39 mM precursor solution was added to all reaction sites, and fluorination was carried out in parallel, followed by CLI imaging (**Figure S1B**).

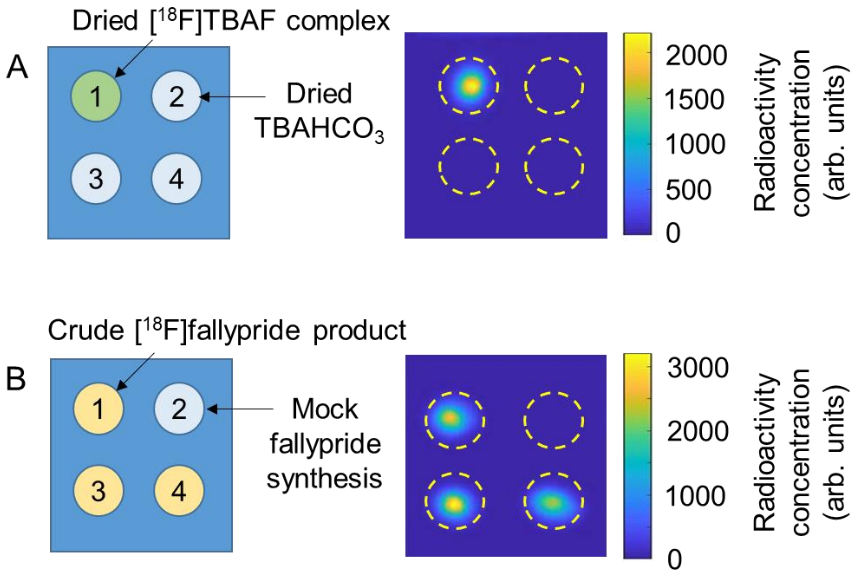

Figure S1: Cross-contamination tests using 2x2 chips. (A) (Left) Schematic of an experiment to test cross-contamination during drying of [^{18}F]TBAF complex. (Right) Cerenkov image of the chip after the drying step. (B) (Left) Schematic of experiment to test cross-contamination during synthesis of [^{18}F]Fallypride. (Right) Cerenkov image of the chip after the fluorination step.

To assess the reproducibility at different reaction sites, syntheses of [^{18}F]fallypride were performed in parallel at multiple sites of the same chip. In a set of experiments on 2x2 chips, we performed drying of the [^{18}F]TBAF complex and subsequent fluorination of tosyl-fallypride on all sites. The crude product was collected from each site and analyzed (**Table S1**).

Table S1: Performance of [^{18}F]Fallypride synthesis on 4 sites on a 2x2 reaction chip. Reactions were carried out with 240 nmol of $TBAHCO_3$, 39 mM of precursor, and 6 µL of precursor solution loaded on each reaction site. High reproducibility is evident.

Performance measure	Reaction site 1	Reaction site 2	Reaction site 3	Reaction site 4	Average ± std dev (n=4)
Collection efficiency (%)	91	92	93	91	92 ± 1
Fluorination efficiency (%)	97	93	94	95	95 ± 2
Crude RCY (%)	89	86	88	87	88 ± 1

We also tested the 4x4 chips to determine if the closer spacing had an impact on cross-contamination. Two experiments were performed with different solution loading patterns. One pattern was made by loading an 8 µL droplet of [^{18}F]fluoride solution (~ 3.6 MBq) on the reaction sites at the four corners of the 4 x 4 array, and another pattern was made by loading an 8 µL droplet of [^{18}F]fluoride solution on alternating reaction sites. The remaining reaction sites were each filled with an 8 µL droplet of DI water. Then, the chips were dried at 100 °C for 1 min, followed by CLI imaging (**Figures S2A** and **S2B**, respectively).

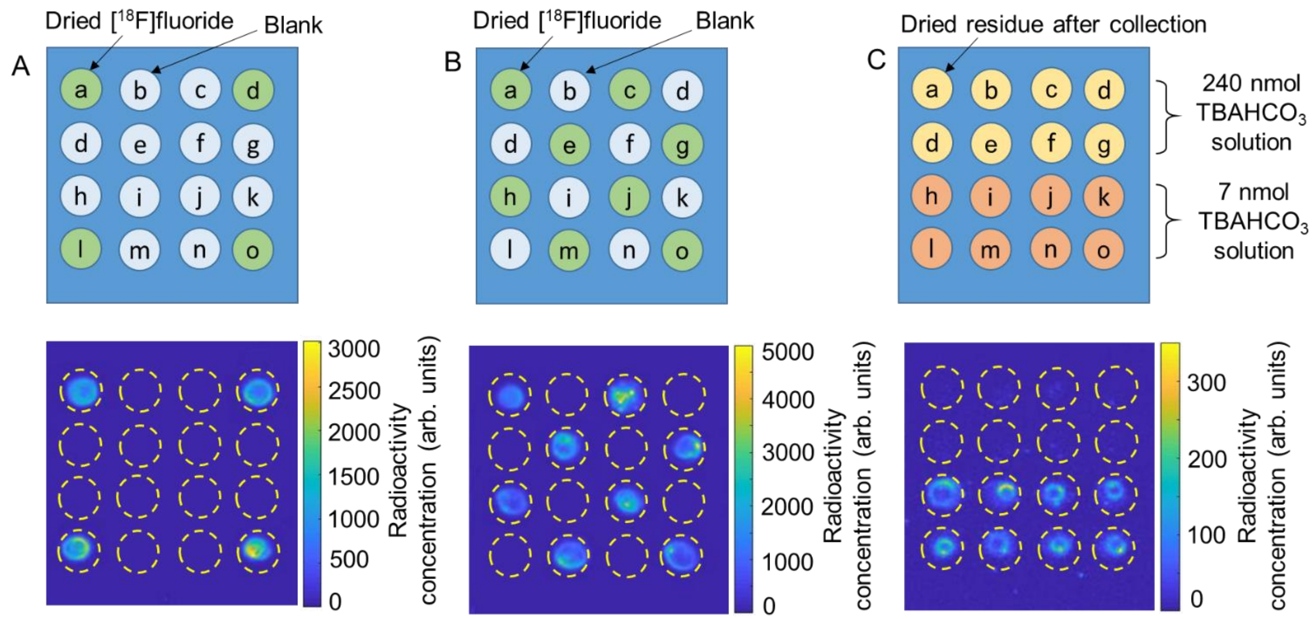

Figure S2: Cross-contamination and repeatability tests using 4x4 chips. (A) (Top) Schematic of an experiment with a pattern of loaded and blank reaction sites. (Bottom) Cerenkov image of the chip after drying. (B) (Top) Schematic of an experiment with a different pattern of loaded and blank reaction sites. (Bottom) Cerenkov image of the chip after drying. (C) (Top) Schematic of experiment to test repeatability during synthesis of [^{18}F]Fallypride (240 nmol $TBAHCO_3$ amount for top two rows and 7 nmol $TBAHCO_3$ amount for bottom two rows). (Bottom) Cerenkov image of the chip after the collection step.

To assess repeatability on the 4x4 chip, we loaded 8 µL of two different concentrations of [^{18}F]fluoride/$TBAHCO_3$ solution: ~3.6 MBq [^{18}F]fluoride and 30 mM [240 nmol] $TBAHCO_3$ in the first 2 rows and ~3.6 MBq [^{18}F]fluoride and 0.9 mM [7 nmol] $TBAHCO_3$ in the second two rows. After the drying step was performed, 6 µL of 39 mM precursor was added to all reaction sites and the fluorination reaction was performed by heating the whole chip. The crude products were collected and analyzed (**Table S2**) and a CLI image of the chip after sample collection was obtained (**Figure S2C**).

Table S2. Synthesis performance from 16 sites on a 4x4 reaction chip using two different base concentrations (n=8 each) corresponding to **Figure S2C**. For all reactions, precursor concentration was 39 mM, and volume of precursor solution was 6 µL. $TBAHCO_3$ amount was 240 nmol in the reactions of rows 1 and 2 on the chip, and 7 nmol in rows 3 and 4. High reproducibility is evident. The higher variability in rows 3 and 4 may be caused by the higher sensitivity to salt concentration under this condition.

	Performance measure	Column 1	Column 2	Column 3	Column 4	Average ± std dev (n=4)
Row 1	Collection efficiency (%)	93	92	94	94	93±1
	Fluorination efficiency (%)	93	92	93	90	92±1
	Crude RCY (%)	87	84	87	84	86±2
Row 2	Collection efficiency (%)	92	95	92	93	93±2
	Fluorination efficiency (%)	89	91	91	89	90±1
	Crude RCY (%)	81	86	84	83	84±2
Row 3	Collection efficiency (%)	92	84	89	88	89±3
	Fluorination efficiency (%)	50	41	41	40	43±5
	Crude RCY (%)	46	35	36	35	38±5
Row 4	Collection efficiency (%)	91	86	88	95	90±4
	Fluorination efficiency (%)	41	45	39	44	42±3
	Crude RCY (%)	37	39	34	42	38±3

6 Optimization data

This section contains all the raw data that was used to generate the plots in **Figure 3** of the main paper. Experiments to compare the effects of amount of base, precursor solution volume, and precursor solution concentration are summarized in **Tables S3, S4,** and **S5**, respectively.

Table S3. Details of syntheses to evaluate influence of base amount (by varying $TBAHCO_3$ concentration in the [^{18}F]fluoride/$TBAHCO_3$ stock solution) on the synthesis of [^{18}F]Fallypride. Reactions were carried out with 77 mM of precursor, 4 µL of precursor solution, and base amount in an 8 µL droplet as indicated. Each condition was replicated n=2 times.

Base amount (nmol)	Collection efficiency %	Fluorination efficiency %	Crude RCY %
480	94 ± 1	63 ± 11	59 ± 9
240	94 ± 1	98.9 ± 0.2	92 ± 1
120	93 ± 1	96 ± 1	88.3 ± 0.3
80	92 ± 1	94 ± 1	86.0 ± 0.2
60	91 ± 3	92 ± 2	83.1 ± 0.5
30	90.4 ± 0.1	65 ± 2	59 ± 2
15	91 ± 2	13 ± 1	12 ± 1
7	88 ± 1	9 ± 1	8 ± 1

Table S4. Details of syntheses to evaluate the influence of precursor solution volume on the synthesis of [^{18}F]Fallypride. Reactions were carried out with 240 nmol of $TBAHCO_3$ in an 8 µL droplet with [^{18}F]fluoride, 77 mM of precursor, and volume of precursor solution as indicated. Each condition was replicated n=2 times.

Precursor solution volume (µL)	Collection efficiency %	Fluorination efficiency %	Crude RCY %
2	87 ± 4	51 ± 7	43 ± 3
4	92 ± 1	96 ± 1	88 ± 2
6	92 ± 1	98 ± 1	90 ± 1
8	92 ± 1	96 ± 1	88 ± 1

Table S5. Details of syntheses to evaluate influence of precursor concentration on the synthesis of [^{18}F]Fallypride. Reactions were carried out with 240 nmol $TBAHCO_3$ in an 8 µL droplet with [^{18}F]fluoride, 6µL of precursor solution, and precursor concentration as indicated. Each condition was replicated n=2 times.

Precursor concentration (mM)	Collection efficiency %	Fluorination efficiency %	Crude RCY %
77	92 ± 2	97 ± 2	89 ± 4
38.5	91 ± 2	96.0 ± 0.5	87 ± 3
19.3	91.1 ± 0.5	81.1 ± 0.3	74 ± 1
9.6	91 ± 1	63 ± 1	57.0 ± 0.5
4.8	89 ± 1	37 ± 1	33 ± 1
2.4	88 ± 2	22 ± 2	19 ± 2
1.2	82 ± 1	13 ± 1	10.4 ± 0.1
0.6	82 ± 4	7 ± 1	5.1 ± 0.5

The data in **Figure S3** shows the correlation between base:precursor ratio and the appearance of radioactive side-products in the crude product.

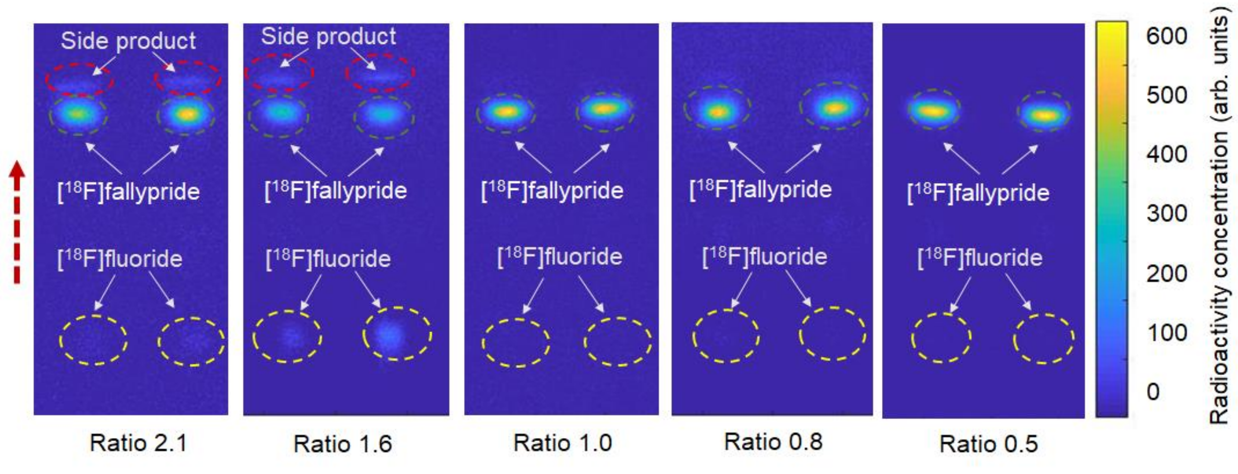

Figure S3. Cerenkov luminescence images of developed TLC plates spotted with 1 µL samples of crude product from microdroplet synthesis of [^{18}F]Fallypride reacted under different base:precursor (molar) ratios. It is evident that higher base:precursor ratios result in greater formation of side product. The dashed circles represent the ROIs for analysis. The dashed arrow represents the direction of solvent flow during developing.

7 HPLC chromatogram of crude [^{18}F]fallypride

Radiochemical purity of the crude sample was determined using analytical-scale radio HPLC (**Figure S4**). Due to the low reagent mass used in droplet synthesis, the chromatogram is very clean. The absence of impurities near the [^{18}F]fallypride peak suggests that purification via analytical-scale radio-HPLC should be straightforward.

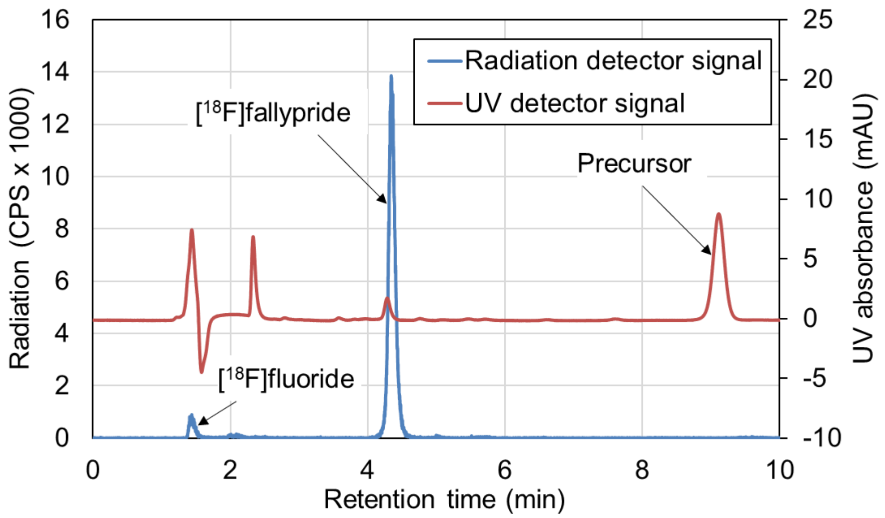

Figure S4. Example of analytical radio-HPLC chromatogram showing the crude [^{18}F]fallypride product synthesized on the microdroplet chip. Non-radioactive fallypride reference standard was injected together with the crude product to confirm the identity of [^{18}F]fallypride.

8 Comparison to macroscale synthesis

In **Table S6**, we compare the optimal conditions found using our microdroplet chip with conditions reported for macroscale syntheses (which are likely based on a limited amount of optimization).

While we expect there to be some differences between the microscale and macroscale due to the different reaction volume, geometry, and heat transfer properties, we anticipate that general trends learned at the microscale could be directly adapted to macroscale apparatus, and the droplet platform may thus be a useful tool to guide optimization of macroscale synthesis. Alternatively, we have recently shown that a fluoride concentrator[4] can be used to significantly increase the amount of activity loaded into a droplet synthesizer, and thus we envision that production-scale synthesis could also be performed in a droplet reactor instead of switching to a conventional macroscale apparatus.

One apparent difference between microscale and macroscale conditions is that the precursor concentration is 5-10x higher for the microscale reaction (though the total amount of precursor is still 16-32x less). However, this may not represent a real difference in optimal conditions: it is likely that higher precursor concentration

would also improve the macroscale reaction, but additional factors are typically considered during macroscale optimization, e.g. minimization of precursor amount (at the expense of reduced yield) to lower the cost of the synthesis and to simplify the purification process. Radiosyntheses in microdroplets are not subject to the same additional constraints, and the microscale apparatus may therefore enable exploration of reaction conditions that are not practical at the macroscale.

Table S6. Comparison of optimal conditions for microdroplet reaction compared to conditions reported for macroscale syntheses.

**Fluorination Reaction Parameter**	**This work**	**Moon et al.**[5]	**Lazari et al.**[6]	**Zhang et al.**[7]
Solvent	1:1 (v/v) thexyl alcohol and MeCN	MeCN	7:3 (v/v) thexyl alcohol and MeCN	MeCN
Volume (µL)	6	1000	1000	1000
Precursor amount (nmol)	230 [0.12 mg]	3900 [2 mg]	7744 [4 mg]	3900 [2 mg]
Precursor concentration in reaction volume (mM)	39	3.9	7.7	3.9
$TBAHCO_3$ amount (nmol)	240 [0.07 mg]	13200 [4.0 mg] or 16500 [5.1 mg] †	15000 [4.6 mg]	7990 [0.8 mg]‡
$TBAHCO_3$ concentration in reaction volume (mM)	40	13.2 or 16.5†	15	7.99
Temperature (°C)	110	100	105	100
Time (min)	7	10	7	10
Isolated RCY (%)	78*	68 ± 2 (n=42)	66 ± 8 (n=6)	82

* Even though the purification is not performed in this study, we have shown in recent work that the optimal conditions found in this work, when combined with a purification step, lead to an isolated radiochemical yield of 78%[8].

†The reported amount is ambiguous, namely 10 µL of 40% $TBAHCO_3$. Values are calculated assuming 40% w/v solution or 40% v/v solution, respectively.

‡ The optimal conditions used $KHCO_3$ instead of $TBAHCO_3$ as a base.

9 References

1 J. Wang, P. H. Chao, S. Hanet and R. M. van Dam, *Lab. Chip*, 2017, **17**, 4342–4355.

2 A. A. Dooraghi, P. Y. Keng, S. Chen, M. R. Javed, C.-J. "CJ" Kim, A. F. Chatziioannou and R. M. van Dam, *Analyst*, 2013, **138**, 5654–5664.

3 J. S. Cho, R. Taschereau, S. Olma, K. Liu, Y.-C. Chen, C. K.-F. Shen, R. M. van Dam and A. F. Chatziioannou, *Phys. Med. Biol.*, 2009, **54**, 6757–6771.

4 P. H. Chao, M. Lazari, S. Hanet, M. K. Narayanam, J. M. Murphy and R. M. van Dam, *Appl. Radiat. Isot.*, 2018, **141**, 138–148.

5 B. Seok Moon, J. Hyung Park, H. Jin Lee, J. Sun Kim, H. Sup Kil, B. Se Lee, D. Yoon Chi, B. Chul Lee, Y. Kyeong Kim and S. Eun Kim, *Appl. Radiat. Isot.*, 2010, **68**, 2279–2284.

6 M. Lazari, J. Collins, B. Shen, M. Farhoud, D. Yeh, B. Maraglia, F. T. Chin, D. A. Nathanson, M. Moore and R. M. van Dam, *J. Nucl. Med. Technol.*, 2014, **42**, 203–210.

7 X. Zhang, R. Dunlow, B. N. Blackman and R. E. Swenson, *J. Label. Compd. Radiopharm.*, 2018, **61**, 427–437.

8 J. Wang, P. H.-S. Chao and R. M. van Dam, *Lab. Chip*, , DOI:10.1039/C9LC00438F.
